# Duolingo-inspired pretesting with words and pictures improves vocabulary learning

**DOI:** 10.1186/s41235-026-00708-y

**Published:** 2026-03-06

**Authors:** Tabitha J. E. Chua, Steven C. Pan

**Affiliations:** Department of Psychology, Faculty of Arts and Social Sciences, National University of Singapore, 9 Arts Link, Singapore City, 117572 Singapore

**Keywords:** Pretesting, Errorful generation, Language learning, Vocabulary, Visual memory

## Abstract

Contemporary language learning applications such as Duolingo and Rosetta Stone often introduce vocabulary through guessing-with-feedback exercises in which learners match words and pictures. We investigated whether that process might yield a *pretesting effect*—that is, the phenomenon where guessing with correct answer feedback (pretesting) enhances memory. Across four experiments, adult online learners engaged in multiple-choice pretesting to learn Spanish word translations shown in word–image (Experiments 1–2) or image–word (Experiments 3–4) format. Relative to a read-only condition, pretesting yielded statistically significant performance improvements on subsequent cued recall (Cohen’s *d* = 0.18–0.40) and, in most cases, multiple-choice tests (*d* = 0.25–0.67), regardless of whether test formats were separately presented or intermixed. Participants also reported preferring pretesting over reading for learning second-language vocabulary, especially for word–image learning. Together, these findings extend the pretesting effect to visual and verbal materials, offering theoretical insights and substantiating word–image and image–word guessing-based approaches of language learning.

**Supplementary Information:**

The online version contains supplementary material available at 10.1186/s41235-026-00708-y.

## Significance statement

When learning a second language, many individuals now turn to modern language learning applications such as Duolingo and Rosetta Stone. These applications offer guessing exercises involving words and pictures, in which users either see a second-language word and guess the corresponding image, or see an image and guess the corresponding word. The correct answer is then shown as feedback. Are these exercises effective for learning vocabulary? Research on the *pretesting effect*—the memory benefit of taking practice tests on unlearned material and receiving feedback on the correct answer—suggests they might. It has been unclear, however, whether this effect extends to the word-and-picture guessing exercises commonly found in language learning apps. Moreover, the theoretical mechanisms by which pretesting might benefit learning in such circumstances remain to be clearly established. In the present study, adults with no prior knowledge of Spanish learned vocabulary words via word–image or image–word guessing exercises. With word–image learning, they either selected the image matching a Spanish word (pretesting) or studied a word–image pair (reading). With image–word trials, they either selected the correct word for an image (pretesting) or studied an image–word pair (reading). Correct answer feedback was always provided. On subsequent cued recall and multiple-choice tests, participants typically performed better on words learned through pretesting, demonstrating that pretesting effects can occur through guessing activities involving words and pictures.The present findings have important theoretical and practical implications: They extend research on the pretesting effect to visual–verbal materials, plus provide evidence that the guessing exercises used in popular language learning applications can support effective vocabulary learning. Moreover, these findings raise the possibility that pretesting effects can emerge in cases where learners lack prior knowledge and preexisting cue-target associations, which are consistent with theoretical accounts of the pretesting effect which suggest that pretesting enhances attention and encoding of correct answers, rather than solely relying upon activating prior knowledge.

**Supplementary Information:**

The online version contains supplementary material available at 10.1186/s41235-026-00708-y.

## Introduction

**Fig. 1:**
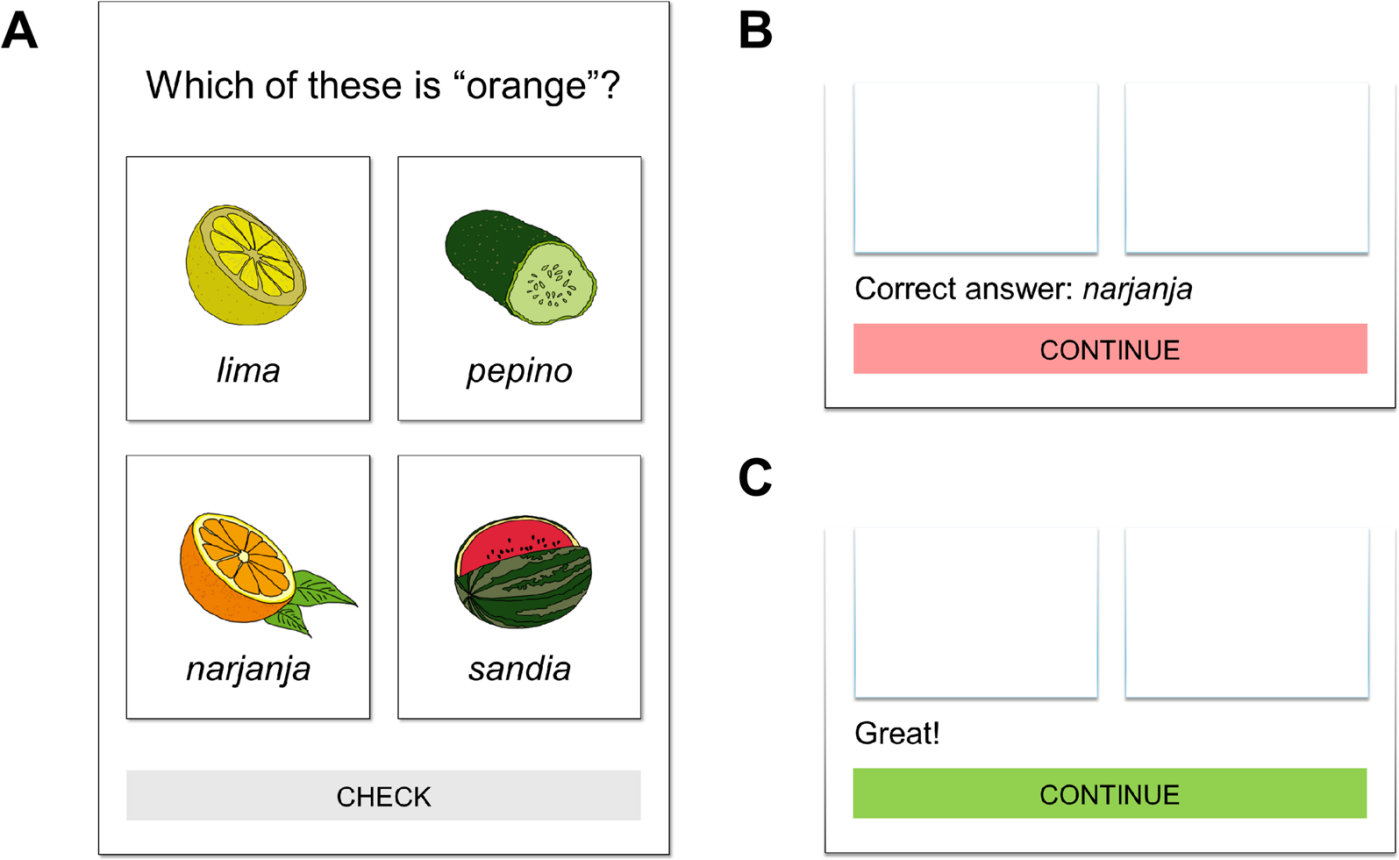
Example of guessing with feedback in a language learning app. Note: Reproduction of a guessing-with-feedback trial in a language learning app that introduces a Spanish vocabulary word. (A) Learners first guess the Spanish word using images and words. (B) Correct answer feedback is provided after an incorrect guess, or (C) correctness feedback is provided after a correct guess. The present study involved a similar guessing procedure but was simplified such that learners guessed from images or words and correct answer feedback always followed

Modern language learning applications often teach vocabulary through guessing-with-feedback exercises that match words and images. For example, Duolingo, the world’s most downloaded language learning application (Sakalauskė & Leonavičiūtė, 2022), teaches words through matching tasks in which users must guess the correct image for a word, the correct word for an image, or guess both words and images (Nushi & Eqbali, 2017). An example of such a task is depicted in Fig. 1. Similarly, Rosetta Stone, another popular application, relies heavily on multiple-choice image-based guessing activities (Rosetta Stone, 2025). Millions of learners now engage in these tasks regularly (Duolingo, 2024). Yet, despite developers’ claims of drawing upon research-backed instructional approaches (Freeman et al., 2023), it remains unclear whether guessing-with-feedback activities involving words and images promote effective vocabulary learning. The present study investigated this issue.

Promisingly, the visual–verbal guessing exercises in language learning applications resemble *pretesting*, a learning technique in which learners take practice tests on unlearned material, often guessing incorrectly, followed by corrective feedback. Doing so can produce a *pretesting effect*, in which memory for pretested information exceeds that from non-guessing methods such as reading or studying (Chan et al., 2018; Kornell & Vaughn, 2016; Pan & Carpenter, 2023; St. Hilaire et al., 2024). Most research to date on pretesting, however, has focused on verbal stimuli (e.g., Kornell et al., 2009; Pan et al., 2019), leaving materials of the type used in such applications largely unexamined (cf. Kang et al., 2013; McGillivray & Castel, 2010). In these studies, pretesting benefits for second-language (L2) learning are sometimes limited (although see Alzahrani et al., 2023; Strong et al., 2025). Whereas robust pretesting benefits on subsequent cued recall tests tend to arise when cues and targets are semantically related and learners possess some prior knowledge (e.g., *doctor–nurse*; Kornell et al., 2009), they are typically absent for L2 word translations or obscure word–definition pairs when learners lack prior semantic knowledge or familiarity with their constituent terms (e.g., *hispid–bristly*; Potts & Shanks, 2014). Benefits of pretesting for such materials, however, sometimes appear on recognition tests (e.g., Butowska et al., 2022; Seabrooke et al., 2021).

The foregoing patterns reflect a key theoretical distinction regarding the pretesting effect, namely whether prior knowledge and preexisting semantic relationships between cues and targets are necessary. Among accounts which maintain that both conditions are essential, the semantic mediator account (Carpenter, 2011) proposes that pretesting activates mediator words linking cues and targets, whereas the search set account (Grimaldi & Karpicke, 2012) suggests that it activates candidate targets, ultimately strengthening the correct answer. In contrast, error correction and error prediction accounts argue that making an incorrect guess—or encountering a mismatch between guess and feedback—enhances attention and encoding without strictly requiring both conditions (i.e., prior knowledge and preexisting semantic relations) to be present (e.g., Potts & Shanks, 2014). Given that L2 word pairs do not meet both conditions, the former accounts explain failures of pretesting to improve cued recall for such word pairs, whereas the latter accounts—who do not require such conditions for a pretesting effect to occur—better accommodate benefits for recognition. Hence, investigating the pretesting effect in L2 learning can provide insights into how purported mechanisms for the effect operate under circumstances that differ from those typically studied in native-language contexts.

When pretesting occurs on words and images, the involvement of visual memory may engage mechanisms distinct from purely verbal tasks. Unlike verbal memory, which relies on semantic encoding (Craik & Lockhart, 1972), visual memory has greater capacity and durability (Brady et al., 2008; Standing, 1973), involves different neural systems (Norman, 2002; Wagner et al., 1998), and relies more on perceptual features (Paivio, 1991). Images are often remembered better than words (Nelson et al., 1976; Standing et al., 1970) and can be recognized accurately even after brief exposures or under varying conditions (Cox & DiCarlo, 2008; Potter & Levy, 1969). These patterns apply to photographs (Potter & Levy, 1969), images of isolated objects (Brady et al., 2008) and, most relevant for the present purposes, simple line drawings (Nelson et al., 1976). Hence, pretesting with visual–verbal L2 materials might engage both visual and verbal systems, enhancing encoding of correct responses (error correction theory) or other productive learning processes, and thereby increasing the likelihood of a pretesting effect on cued recall and/or recognition-type tests. Alternatively, if the pretesting effect requires prior semantic relationships and knowledge, benefits may remain minimal given that verbal–visual L2 word pairs typically lack both.

To investigate whether the guessing-with-feedback exercises in L2 learning applications produce pretesting effects, we conducted four experiments involving Spanish vocabulary words presented in verbal–visual (word–image; Experiments 1–2) or visual–verbal (image–word; Experiments 3–4) formats. In each experiment, participants learned pairs through multiple-choice pretesting or reading and then completed cued recall and multiple-choice tests. To assess robustness and potential boundary conditions, Experiments 1 and 3 intermixed cued recall and multiple-choice trials, whereas Experiments 2 and 4 varied trial presentation (intermixed vs. blocked). We also collected metacognitive judgments to compare perceptions of pretesting and reading.

## Experiment 1

Experiment 1 investigated the effects of pretesting versus reading for word**–**image learning, using a format similar to that used with Duolingo and Rosetta Stone.

### Method

This experiment was preregistered at https://aspredicted.org/2vnm-xr86.pdf.

#### Participants

An a priori G*Power analysis (Faul et al., 2007) indicated that 35 participants would provide 80% power to detect a pretesting effect of *d* = 0.49 at α = .05 (cf. Potts & Shanks, 2014). We recruited 78 adult learners from Prolific Academic (USD $2.67 compensation each), all meeting the eligibility criteria of English fluency, residence in an English-speaking country, ≥ 95% Prolific approval, and aged 21–45. Twenty participants were excluded for prior Spanish knowledge, comprehension check failures, or non-completion, leaving 58 participants (*M*_age_ = 33.7, 47% female). The entire study received ethics approval, and informed consent was obtained beforehand.

#### Materials

The materials included 36 Spanish nouns, 36 target images, 108 distractor images (word–image; Experiments 1–2), and 108 distractor nouns (image–word; Experiments 3–4) (all archived at https://osf.io/s8gvp/). Except two hand-drawn items created in the same style, all images came from Multipic (Duñabeitia et al., 2018), Linguapix (Krautz & Keuleers, 2022), and IPNP (Szekely et al., 2005). Each noun denoted a concrete, familiar object, differed visibly from its English equivalent, and was under 10 letters in Spanish and English.

The nouns included two examples each of 18 common object categories—accessories, anatomy, animals, clothing, entertainment, furniture, gear, gifts, household, jobs, nature, notebooks, parks, outerwear, stationery, tools, utensils, vehicles, and food. These nouns were divided into four sub-lists, each containing one noun from nine categories, with counterbalanced assignment across the learning phase (18 pretested or read) and test phase (half cued recall, half multiple-choice). Sub-lists were matched on mean word frequency (0–127) and concreteness (5.28–7) using norms from Nelson et al. (2004).

#### Design and procedure

**Fig. 2:**
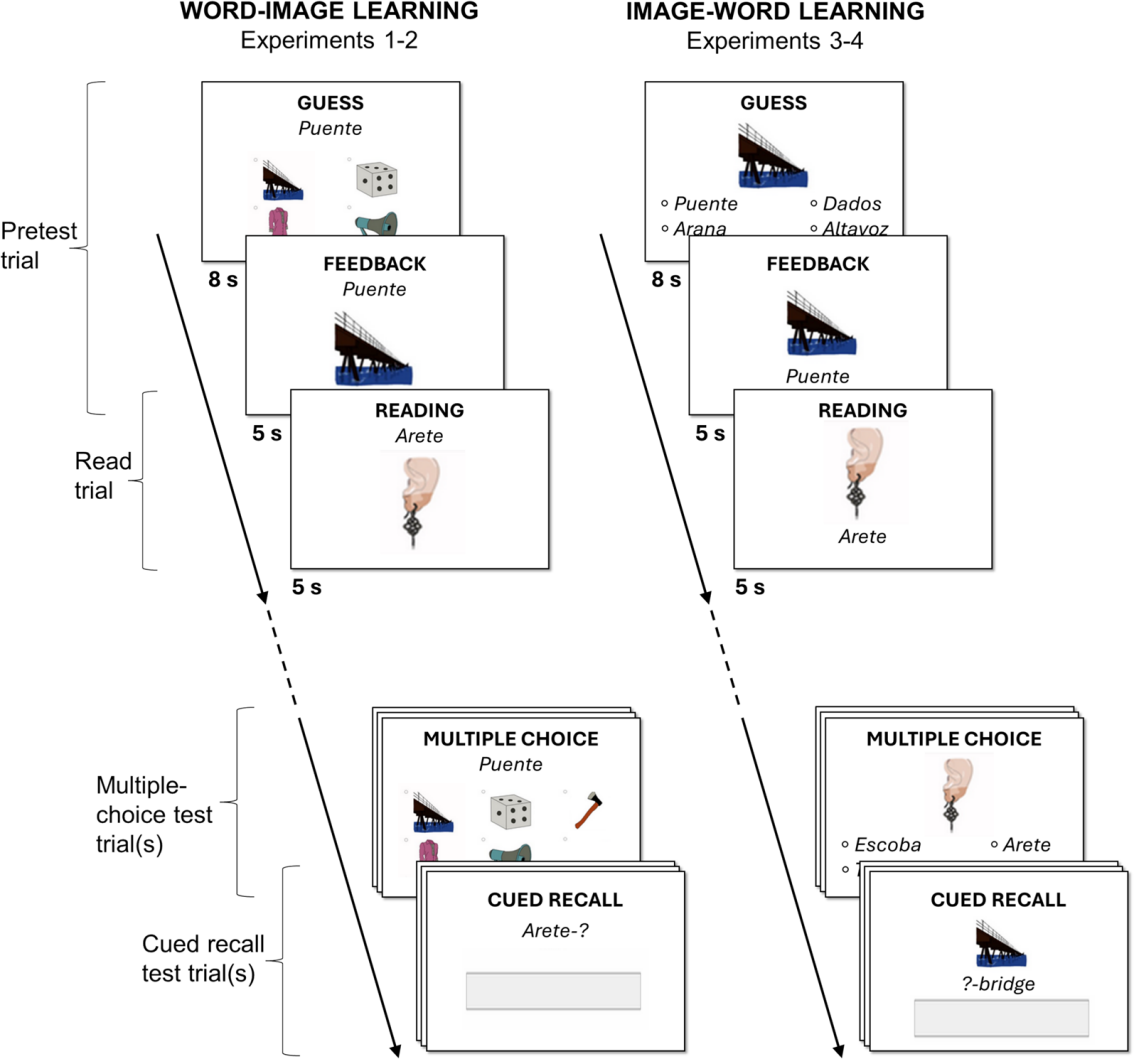
Word–image and image–word learning procedures. Note. Shown are examples of a pretesting and a reading trial (from the *learning phase*), as well as a multiple-choice and cued recall trial (from the *criterial test phase*). Assignment of stimulus items to pretesting or reading, multiple-choice or cued recall, and order of trials were all randomized (except for the blocked groups of Experiments 2 and 4, where participants received all multiple-choice test trials before cued recall test trials, or vice versa)

The procedure is depicted in the left-side portion of Fig. 2. There were two independent variables: Learning Condition (pretesting vs. reading) and Criterial Test Format (cued recall vs. multiple choice), both of which were within-subjects.

*Learning Phase*: Participants first learned the meaning of 36 Spanish nouns, one at a time, through pretesting or reading trials (randomly intermixed). Similar to approaches taken in language learning applications, each word was paired with a corresponding image. For pretesting, participants saw a Spanish word and guessed its meaning from four images, one of which was the correct answer. They had 8 s to guess and 5 s to view correct answer feedback (similar to Knight et al., 2012; Kornell et al., 2009, and others). For reading, participants studied the word–image pair directly for 5 s. Doing so equated the time spent viewing the Spanish word paired with its definition in image form; it is acknowledged, however, that participants in the pretesting condition had longer exposure to the Spanish word alone, presumably without knowing its correct meaning. In prior research with purely verbal materials, providing the reading condition extra time to view both the cue and target together can attenuate—but not reverse—the advantages of pretesting (e.g., Kornell et al., 2009).

*Distractor Task*: Participants completed five one-minute category knowledge tasks (e.g., listing movies).

*Criterial Test:* Memory was assessed via cued recall and multiple-choice tests, approximating scenarios wherein L2 learners generate a translation from memory or identify it among alternatives, respectively. Participants saw each Spanish word, one at a time, and defined it in English (cued recall) or selected its meaning from six images (one target image, three of the same distractors as during the learning phase, and two distractors from a different cue). Trials were self-paced, with cued recall and multiple-choice trials randomly intermixed.

*Metacognitive Questions*: After the criterial test, participants answered a series of metacognitive questions, including relative effectiveness and preference for pretesting versus reading, as well as postdictions of test performance (results are discussed after presentation of the final experiment).

#### Data analysis

Null hypothesis significance testing (NHST) was conducted with *α* = .05, and 95% confidence intervals (CIs) are reported where applicable (Cumming, 2014). Bayes factors (*BF*_*10*_) were computed for all pairwise comparisons (Rouder & Morey, 2012). A *BF*_*10*_ > 1 indicates evidence for the alternative hypothesis; when *BF*_*10*_ < 1, we report the reciprocal *BF*_*01*_ to quantify evidence in favor of the null. Across experiments, some violations of normality in the lower tail were observed, prompting nonparametric tests which showed the same patterns as the parametric results (see Supplementary Materials).

In some pretesting studies, correctly guessed items are excluded (e.g., Huelser & Metcalfe, 2012; Kornell et al., 2009), but in the present experiments, such items were more frequent. The analyses reported here were conducted on criterial test data for all items (see Supplementary Materials for analyses conditionalized on guessing accuracy).

### Results

#### Pretest performance

Guessing performance for all experiments is presented in Table 1. Accuracy was modestly above chance, indicating minimal prior knowledge.

**Table 1:** Pretest performance

Learning context	Experiment	Mean (*SD*)
Word–image learning	1	0.38 (.15)
	2, intermixed cued recall and multiple-choice test trials	0.36 (.15)
	2, blocked cued recall and multiple-choice test trials	0.35 (.14)
Image–word learning	3	0.36 (.13)
	4, intermixed cued recall and multiple-choice test trials	0.38 (.14)
	4, blocked cued recall and multiple-choice test trials	0.35 (.17)

Pretest performance refers to the proportion of correctly guessed images (Experiments 1 and 2) or words (Experiments 3 and 4) during the multiple-choice pretest

#### Criterial test performance

**Fig. 3:**
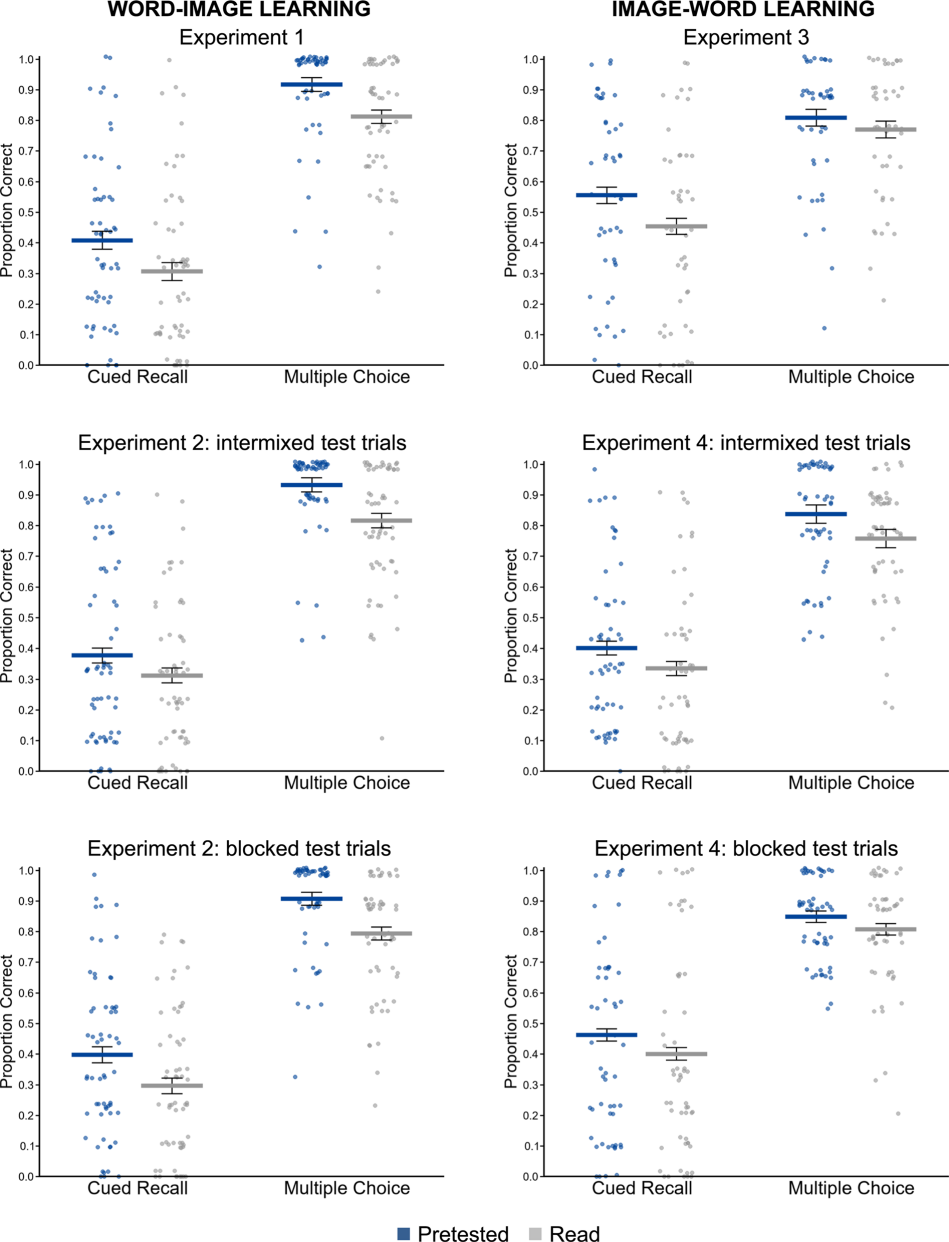
Criterial test results. Note. Each panel depicts criterial test performance separately for cued recall and multiple-choice items that were previously pretested and previously read. The horizontal bars represent means, whereas the error bars represent standard errors of the within-subject difference scores between the pretested and read conditions. The scatterplots represent subject-level means

Results for all word–image learning experiments are shown in Fig. 3 (left-side panels).

*Cued Recall*: Pretesting produced a statistically significant advantage over reading (*M* = .41 vs. .31), *t*(57) = 3.46, *p* = .001, *d* = 0.36, 95% CI [0.15, 0.58], *BF*_*10*_ = 26.42. (Note: intrusions—associative errors in which a studied term was recalled in response to the wrong cue, rather than the cue it was originally paired with (e.g., recalling *ball* instead of *egg* in response to an image of an egg)—were generally rare, constituting 13% and 14% of all responses in the pretested and read conditions, respectively; similar or lower analogous intrusion rates were observed in all subsequent experiments, with no apparent differences across conditions, and are not addressed further).

*Multiple Choice*: Pretesting produced a statistically significant advantage over reading (*M* = .92 vs. .81), *t*(57) = 4.73, *p* < .001, *d* = 0.59, 95% CI [0.32, 0.86], *BF*_*10*_ = 1254.13.

## Experiment 2

Experiment 1 demonstrated that pretesting can improve word–image learning as measured on cued recall and multiple-choice tests. We next sought to replicate this effect under conditions where cued recall and multiple-choice trials are not mixed together, which might reduce contextual retrieval cues (cf. Hirshman & Bjork, 1988; Mulligan & Peterson, 2008). This pattern was motivated by pilot tests in which we adjusted the difficulty of the distractors used on multiple-choice test trials and observed changes in cued recall performance, raising the prospect that answer choices presented on multiple-choice trials might facilitate or inhibit participants’ ability to reconstruct memories of the stimuli presented on adjacent cued recall trials. Moreover, we noted that mixing test formats does not necessarily occur in language learning applications and is relatively uncommon in pretesting studies (e.g., Potts & Shanks, 2014).

### Method

This experiment was preregistered at https://aspredicted.org/8qgm-m66w.pdf.

#### Participants

Participants were randomly assigned to intermixed or blocked groups. A target sample of 100 participants (50 per group) was determined via an a priori power analysis using the *Superpower* package in R (Caldwell et al., 2020) and Experiment 1 data, assuming a cued recall pretesting effect of 0.10 for intermixed trials and zero for blocked trials. This sample size provided > 80% power to detect a Learning Condition × Trial Arrangement interaction at *α* = .05. A total of 153 participants were recruited from Prolific in the same manner as Experiment 1; after excluding those with prior Spanish knowledge, comprehension failures, or off-task behavior, 63 remained in the intermixed group (*M*_age_ = 32.8, 56% female) and 60 in the blocked group (*M*_age_ = 32.8, 55% female).

#### Design, materials, procedure, and data analysis

All aspects were drawn from Experiment 1, except that participants were randomly assigned to a criterial test in which cued recall and recognition trials were intermixed or shown in separate contiguous blocks, with block order randomized. In summary, there were two independent variables: Learning Condition (pretesting vs. reading; within-subjects) and Trial Arrangement (blocked vs. intermixed; between-subjects).

### Results

#### Cued recall

A 2 × 2 ANOVA revealed a significant main effect of Learning Condition, *F*(1, 121) = 22.14, *p* < .001, *η*_*p*_^*2*^ = .150, but no effect of Trial Arrangement, *F*(1, 121) = 0.00, *p* = .957, *η*_*p*_^*2*^ < .001, nor interaction, *F*(1, 121) = 1.06, *p* = .305, *η*_*p*_^*2*^ = .009, suggesting that trial arrangement is inconsequential—that is, mixing multiple-choice and cued recall trials is not necessary for pretesting effects to emerge for cued recall. Pretesting outperformed reading in both the intermixed (*M* = .38 vs. .31, *t*(62) = 2.70, *p* < .01, *d* = .23, 95% CI [.06, .41], *BF*₁₀ = 3.77) and blocked groups (*M* = .40 vs. .30, *t*(59) = 3.91, *p* < .001, *d* = .40, 95% CI [.20, .61], *BF*₁₀ = 97.28), replicating Experiment 1.

#### Multiple choice

A corresponding 2 × 2 ANOVA revealed a significant main effect of Learning Condition, *F*(1, 121) = 52.50, *p* < .001, *η*_*p*_^*2*^ = .300, but no effect of Trial Arrangement, *F*(1, 121) = 0.815, *p* = .368, *η*_*p*_^*2*^ = .007, nor interaction, *F*(1, 121) = 0.01, *p* = .914, *η*_*p*_^*2*^ < .001, similar to the cued recall results. Pretesting outperformed reading in both the intermixed (*M* = .93 vs. .82, *t*(62) = 4.95, *p* < .001, *d* = .67, 95% CI [0.38, 0.97], *BF*₁₀ = 2922.50) and blocked groups (*M* = .91 vs. .79, *t*(59) = 5.37, *p* < .001, *d* = .64, 95% CI [0.38, 0.90], *BF*₁₀ = 11,449.76), again replicating Experiment 1.

## Experiment 3

Experiment 3 extended our investigation to a complementary but theoretically and practically important scenario in L2 learning: image–word learning, in which learners must produce or recognize words based on pictures.

### Method

This experiment was preregistered at https://aspredicted.org/6w3c-rfbd.pdf. Although results are reported separately for clarity, Experiments 3–4 were conducted over an overlapping time period with Experiments 1–2.

#### Participants

The target sample size was identical to, and based on the same power analysis as, that of Experiment 1. Sixty-five participants were recruited from Prolific Academic in the same manner as in the prior experiments. Eighteen participants were excluded for prior Spanish knowledge, comprehension check failures, or non-completion, leaving 47 participants in the final sample (*M*_*age*_ = 31.0, 34% female).

#### Design, materials, procedure, and data analysis

All procedures mirrored Experiment 1 except for the change to image–word learning (see right-side portion of Fig. 2). During the learning phase, pretesting involved participants viewing an image and selecting the correct Spanish word from four options, whereas reading involved viewing an image with its corresponding Spanish word. On the criterial test, participants either typed the Spanish word (cued recall) or selected it from four choices (multiple choice)**,** approximating scenarios in which L2 learners either produce a word from memory or recognize it among alternatives. For cued recall, a one-word English label accompanied each image (e.g., “*bridge*” with a bridge photo); we added this feature, which is in line with the approach taken by some language learning applications, after pilot testing.

#### Criterial test performance

Results for all image–word learning experiments are shown in Fig. 3 (right-side panels).

#### Cued recall

Pretesting produced a statistically significant advantage over reading (*M* = .56 vs. .45), *t*(46) = 3.86, *p* < .01, *d* = 0.33, 95% CI [0.16, 0.51], *BF₁₀* = 73.61.

#### Multiple choice

Multiple-choice performance was not significantly different after pretesting versus reading, (*M* = .81 vs. .77), *t*(46) = 1.39, *p* = .173, *d* = 0.18, 95% CI [-0.08, 0.44], *BF₀₁* = 2.59.

## Experiment 4

Experiment 3 demonstrated that pretesting can improve image–word learning as evident on cued recall, but not necessarily multiple-choice, tests. Mirroring the logic of Experiment 2, Experiment 4 investigated the reproducibility of these patterns under varying trial arrangements.

### Method

This experiment was preregistered at https://aspredicted.org/4h8n-qc32.pdf.

#### Participants

Participants were randomly assigned to intermixed or blocked groups, mirroring Experiment 2. The target sample size of 100 participants (50 per group) was chosen to match Experiment 2, relying on the same power analysis (i.e., assuming a 0.10 cued recall effect in the intermixed group, as also occurred in Experiment 3, and zero in the blocked group). Of 162 recruited, exclusions for prior Spanish knowledge, comprehension failures, or off-task behavior left 57 in the intermixed group (*M*_*age*_ = 32.5, 51% female) and 56 in the blocked group (*M*_*age*_ = 32.0, 52% female).

#### Design, materials, procedure, and data analysis

All aspects mirrored Experiment 3, except participants completed cued recall and multiple-choice test trials that were intermixed or in separate blocks, as in Experiment 2. Data analysis followed the same plan as Experiment 2.

### Results

#### Cued recall

A 2 × 2 ANOVA revealed a significant main effect of Learning Condition, *F*(1, 111) = 17.46, *p* < .001, *η*_*p*_^*2*^ = .14, but no effect of Trial Arrangement, *F*(1, 111) = 1.27, *p* = .261, *η*_*p*_^*2*^ = .01, nor interaction, *F*(1, 111) = 0.02, *p* = .876, *η*_*p*_^*2*^ < .001, suggesting that trial arrangement is inconsequential. Pretesting outperformed reading in both blocked (*M* = .46 vs. .40, *t*(55) = 3.04, *p* = .004, *d* = .18, 95% CI [.06, .31], *BF*₁₀ = 8.83) and intermixed groups (*M* = .40 vs. .34, *t*(56) = 2.89, *p* = .005, *d* = .23, 95% CI [.07, .40], *BF*₁₀ = 6.08), albeit with smaller effect sizes than in Experiment 3.

#### Multiple choice

A 2 × 2 ANOVA revealed a significant main effect of Learning Condition, *F*(1, 111) = 11.86, *p* < .001, *η*_*p*_^*2*^ = .10, but no effect of Trial Arrangement, *F*(1, 111) = 1.24, *p* = .268, *η*_*p*_^*2*^ = .01, nor interaction, *F*(1, 111) = 1.17, *p* = .281, *η*_*p*_^*2*^ = .01, similar to the cued recall results. Pretesting outperformed reading in both blocked (*M* = .85 vs. .81, *t*(55) = 2.26, *p* = .028, *d* = .25, 95% CI [.03, .48], *BF*₁₀ = 1.50) and intermixed groups (*M* = .84 vs. .76, *t*(56) = 2.67, *p* = .010, *d* = .44, 95% CI [.10, .78], *BF*₁₀ = 3.61), in contrast with Experiment 3.

## Metacognitive results

As shown in Table 2, participants significantly preferred pretesting over reading for word–image learning (binomial tests, *p* ≤ .030), with similar but nonsignificant trends observed for image–word learning. Overall postdiction judgments were also higher for pretesting than reading in all experiments except Experiment 3, with significant differences in Experiments 1 and 2 (*t* tests, *p* ≤ .027; see Table 3 for full results).

**Table 2:** Comparison of methods (% of respondents)

Type	Experiment	Helped learn better…			Prefer to use in future…		
		Reading	Pretesting	Neither/unsure	Reading	Pretesting	Neither/unsure
Word–image learning	1	31	62*	7	31	62*	7
	2, intermixed	27	56*	17	30	57*	13
	2, blocked	32	60*	8	33	58	9
Image–word learning	3	43	45	12	43	45	12
	4, intermixed	33	56	11	37	51	12
	4, blocked	39	50	11	39	46	15

^*^p < .05. Data represent the percentage of participants, in each experiment, that choose either learning method (or neither/unsure), in response to each of two metacognitive questions administered after the criterial test

**Table 3:** Postdictions, mean (SD)

Type	Experiment	Overall		Cued recall		Multiple choice	
		Reading	Pretesting	Reading	Pretesting	Reading	Pretesting
Word–image learning	1	0.55 (0.23)	0.70 (0.21)**	0.36 (0.27)	0.38 (0.25)	0.46 (0.29)	0.50 (0.25)
	2, intermixed	0.59 (0.26)	0.74 (0.20)**	0.37 (0.28)	0.40 (0.27)	0.48 (0.28)	0.58 (0.24)**
	2, blocked	0.59 (0.24)	0.66 (0.23)	0.30 (0.25)	0.35 (0.25)*	0.51 (0.26)	0.56 (0.26)*
Image–word learning	3	0.67 (0.25)	0.60 (0.23)	0.41 (0.29)	0.41 (0.28)	0.59 (0.21)	0.62 (0.19)
	4, intermixed	0.59 (0.27)	0.68 (0.24)	0.26 (0.22)	0.34 (0.24)**	0.45 (0.25)	0.56 (0.23)***
	4, blocked	0.59 (0.25)	0.63 (0.22)	0.33 (0.30)	0.35 (0.30)	0.56 (0.26)	0.55 (0.27)

^*^p < .05, ** p < .01, *** p < .001. Data represent the estimates that participants gave for their overall criterial test performance, for cued recall items only, and for multiple-choice items only

## Discussion

Across four experiments, pretesting with words and pictures—inspired by guessing-with-feedback exercises common to Duolingo, Rosetta Stone, and similar applications—consistently improved performance on cued recall tests (*d* = 0.18–0.40), regardless of whether word–image or image–word associations were involved and whether trials were intermixed or blocked. Multiple-choice tests also showed significant benefits (*d* = 0.25–0.67) in all cases except Experiment 3. These findings demonstrate that pretesting effects extend to visual–verbal stimuli in L2 learning contexts and, moreover, imply that theoretical mechanisms implicated in such effects—such as error correction and associative strengthening—can operate even when learners have minimal prior knowledge and semantic associations (i.e., such mechanisms can generalize beyond learning in one’s own native language). Remarkably, the benefits appeared to encompass verbal content (Spanish word spellings) and semantic content (word meanings), suggesting that guessing-with-feedback exercises effectively promote vocabulary learning across diverse contexts.

For cued recall, pretesting enhanced participants’ ability to retrieve both Spanish word definitions and corresponding Spanish words from image cues. In the case of word–image learning, participants retrieved the associated image and mentally translated it into English. Pretesting facilitated such cross-modal transfer, possibly by leveraging the high capacity and fidelity of visual memory (Brady et al., 2008) while enabling perceptual details to support translation into verbal recall (cf. Paivio, 1991). Pretesting may have strengthened word–image associations through error correction or other associative processes, improving both image retrieval and subsequent verbal recall.

In image–word learning, participants retrieved Spanish words in response to image cues, aided by an English label. Although more challenging due to unfamiliarity with Spanish words, pretesting still conferred an advantage, albeit smaller in effect size terms than with word–image learning. This advantage may have arisen from error generation processes strengthening links between visual cues and lexical targets or from the English label serving as a mediator or recall prompt. In either scenario, pretesting improved recall of the Spanish word.

For multiple-choice tests, pretesting enhanced selection of the correct image for a given Spanish word, or vice versa, relative to reading. Mechanisms implicated in recognition effects for semantically unrelated verbal pairs—such as error correction or item-specific memory enhancement (Seabrooke et al., 2021)—may therefore apply to visual–verbal learning. The sole exception, Experiment 3, possibly reflects a ceiling effect; modest Bayesian evidence for the null and the significant effects in Experiment 4 indicate that pretesting generally enhances multiple-choice performance, despite occasional variability.

Notably, the cued recall benefits observed here differ from studies of purely verbal L2 word translations (e.g., Butowska et al., 2022), where such effects were absent—a finding that has been interpreted to suggest that prior semantic relationships and knowledge are essential for a pretesting effect to emerge (indeed, the present results suggest that mechanisms implicated in the pretesting effect can circumvent those purported theoretical requirements). Whether these differences arise from the use of visual–verbal materials or other factors unique to the Spanish vocabulary stimuli requires further research. The multiple-choice findings, however, better align with prior results involving purely verbal materials (e.g., Hollins et al., 2023; Potts & Shanks, 2014; Potts et al., 2019).

In contrast with prior studies (e.g., Huelser & Metcalfe, 2012; Pan & Rivers, 2023) and surveys (e.g., Pan et al., 2020), participants viewed pretesting as more effective for learning than reading and preferred to use it in the future, particularly for word–image learning. These patterns could suggest a greater openness to guessing-with-feedback in L2 learning and may indicate that learners can accurately monitor and appreciate the benefits of pretesting when visual–verbal materials are involved. Another possibility involves the relatively high guessing accuracy (35–38% for each experiment); prior studies (Kornell & Bjork, 2007; Tullis et al., 2013; Vaughn & Kornell, 2019) have shown that learners enjoy being tested when they manage to answer correctly.

Future research could clarify factors that may have influenced the present results and address study limitations. Item difficulty is one such factor: the high guessing accuracy raises the possibility that participants may have had prior knowledge of some of the words despite the exclusion criteria of prior Spanish knowledge. Although these patterns do not necessarily create a confound given the equal distribution of correct items across conditions, they raise the prospect that pretesting was occurring on previously learned or somewhat familiar information. It should be emphasized, however, that learning gains were also observed for incorrectly guessed items (see Supplementary Materials), and hence, benefits of pretesting were not necessarily limited to items that participants already knew. Issues for further study also include the mechanisms underlying pretesting effects for visual–verbal materials, the impact of less meaningful or visually discontinuous stimuli, variations in test format (e.g., two-alternative forced-choice), trial timing, replicability under between-subjects design conditions (Slamecka & Katsaiti, 1987), and individual differences in cognitive ability (e.g., Pan et al., 2025). To further isolate the role of visual–perceptual versus verbal details, future work could also manipulate the presence of images versus words.

In conclusion, the present experiments demonstrate that pretesting effects can emerge from guessing-with-feedback exercises involving words and pictures, similar to those used in Duolingo, Rosetta Stone, and comparable applications. These results suggest that mechanisms contributing to pretesting effects can operate even when semantic knowledge is minimal. Our findings therefore affirm the pedagogical value of such exercises for L2 vocabulary learning. Moreover, it seems likely that incorporating pretesting into other language learning contexts can enhance learning and retention compared with conventional reading-based methods.

## Supplementary Information

Additional file1 (DOCX 45 kb)

## Data Availability

Available at: https://osf.io/s8gvp/?view_only=0a1ba4c2784c4f5387535cf10f860deb

## Code availability

Available at: https://osf.io/s8gvp/?view_only=0a1ba4c2784c4f5387535cf10f860deb
