# Supplementary material for: Duolingo-inspired pretesting with words and pictures improves vocabulary learning
Source: Cogn Res Princ Implic. 2026 Mar 6;11:20. doi: 10.1186/s41235-026-00708-y (PMC12965936; doi:10.1186/s41235-026-00708-y)
Supplement: Supplementary file 1 — Additional file1 (DOCX 45 kb) [file 41235_2026_708_MOESM1_ESM.docx]

# Supplementary Online Materials for:

***Duolingo-Inspired Pretesting with Words and Pictures Improves Vocabulary Learning***

This document contains the results of supplementary analyses. Specifically, Table S1 features the results of nonparametric pairwise comparisons conducted on the criterial test results of each experiment; Tables S2 and S3 present descriptive statistics for the cued recall and recognition test results conditionalized by correct or incorrect guessing, respectively; Table S4 details the corresponding pairwise comparisons (Note: interpretation of these patterns is complicated by potential item-selection effects and the inability to classify read items by guessing accuracy).

# Table S1

*Wilcoxon Signed-Rank Test Results*

| Test format | Type | Experiment | *W* | *p-*value |
| --- | --- | --- | --- | --- |
| Cued recall | Word-image learning | 1 | 940.5 | .003** |
|  |  | 2, blocked | 1054.0 | <.001*** |
|  |  | 2, intermixed | 1053.5 | .007** |
|  | Image-word learning | 3 | 410.5 | .001** |
|  |  | 4, blocked | 573.0 | .003** |
|  |  | 4, intermixed | 545.0 | .011* |
| Multiple- | Word-image learning | 1 | 465.0 | <.001*** |
| choice |  | 2, blocked | 687.0 | <.001*** |
|  |  | 2, intermixed | 673.5 | <.001*** |
| Image-word learning | | 3 | 317.5 | .320 |
|  | | 4, blocked | 421.5 | .032* |
|  | | 4, intermixed | 736.0 | .013* |
| *Note. *p* < .05,***p* < .01,****p* < .001. Shown are the results of nonparametric pairwise comparisons (pretested vs. read) for both criterial tests (cued recall and multiple-choice) for each experiment. | | | | |

# Table S2

*Cued Recall Performance for All Items and Conditionalized by Correct or Incorrect Guessing*

Type Experiment Conditionalization % Data Mean (*SD*)

| Word-image | 1 | Pretested items guessed incorrectly | 62 | .37 (.30) |
| --- | --- | --- | --- | --- |
| learning |  | Pretested items, guessed correctly | 38 | .47 (.35) |
|  |  | Pretested items, all | 100 | .41 (.29) |
|  |  | Read items, all | 100 | .31 (.27) |
|  | 2, blocked | Pretested items guessed incorrectly | 62 | .38 (.26) |
|  |  | Pretested items, guessed correctly | 38 | .46 (.38) |
|  |  | Pretested items, all | 100 | .40 (.27) |
|  |  | Read items, all | 100 | .30 (.24) |
|  | 2, intermixed | Pretested items guessed incorrectly | 63 | .33 (.30) |
|  |  | Pretested items, guessed correctly | 37 | .43 (.36) |
|  |  | Pretested items, all | 100 | .38 (.29) |
|  |  | Read items, all | 100 | .31 (.27) |
| Image-word | 3 | Pretested items guessed incorrectly | 66 | .54 (.31) |
| learning |  | Pretested items, guessed correctly | 34 | .60 (.37) |
|  |  | Pretested items, all | 100 | .56 (.30) |
|  |  | Read items, all | 100 | .45 (.31) |
|  | 4, blocked | Pretested items guessed incorrectly | 67 | .42 (.36) |
|  |  | Pretested items, guessed correctly | 33 | .52 (.35) |
|  |  | Pretested items, all | 100 | .46 (.33) |
|  |  | Read items, all | 100 | .40 (.34) |
|  | 4, intermixed | Pretested items guessed incorrectly | 66 | .40 (.32) |
|  |  | Pretested items, guessed correctly | 34 | .42 (.34) |
|  |  | Pretested items, all | 100 | .40 (.27) |
|  |  | Read items, all | 100 | .34 (.29) |

*Note.* Data refers to the percentage of all criterial test data for that condition (pretested or read).

# Table S3

*Multiple-Choice Performance for All Items and Conditionalized by Correct or Incorrect Guessing*

Type Experiment Conditionalization % Data Mean (*SD*)

| Word-image | 1 | Pretested items guessed incorrectly | 61 | .90 (.18) |
| --- | --- | --- | --- | --- |
| learning |  | Pretested items, guessed correctly | 39 | .94 (.16) |
|  |  | Pretested items, all | 100 | .92 (.16) |
|  |  | Read items, all | 100 | .81 (.19) |
|  | 2, blocked | Pretested items guessed incorrectly | 65 | .89 (.18) |
|  |  | Pretested items, guessed correctly | 35 | .97 (.12) |
|  |  | Pretested items, all | 100 | .91 (.15) |
|  |  | Read items, all | 100 | .79 (.19) |
|  | 2, intermixed | Pretested items guessed incorrectly | 61 | .94 (.13) |
|  |  | Pretested items, guessed correctly | 39 | .94 (.19) |
|  |  | Pretested items, all | 100 | .93 (.13) |
|  |  | Read items, all | 100 | .82 (.20) |
| Image-word | 3 | Pretested items guessed incorrectly | 64 | .76 (.25) |
| learning |  | Pretested items, guessed correctly | 36 | .87 (.26) |
|  |  | Pretested items, all | 100 | .81 (.21) |
|  |  | Read items, all | 100 | .77 (.21) |
|  | 4, blocked | Pretested items guessed incorrectly | 64 | .81 (.20) |
|  |  | Pretested items, guessed correctly | 36 | .90 (.20) |
|  |  | Pretested items, all | 100 | .85 (.13) |
|  |  | Read items, all | 100 | .81 (.18) |
|  | 4, intermixed | Pretested items guessed incorrectly | 60 | .83 (.22) |
|  |  | Pretested items, guessed correctly | 40 | .84 (.29) |
|  |  | Pretested items, all | 100 | .84 (.18) |
|  |  | Read items, all | 100 | .76 (.19) |

*Note.* Data refers to the percentage of all criterial test data for that condition (pretested or read).

# Table S4

*Paired t-Test Results Involving Pretested Items Conditionalized by Correct or Incorrect Guessing*

| Conditionalized | Test format | Type | Experiment | *df* | *t* | *p-*value |
| --- | --- | --- | --- | --- | --- | --- |
| Pretested items | Cued recall | Word-image | 1 | 57 | 1.650 | .105 |
| guessed |  | learning | 2, blocked | 59 | 3.071 | .003** |
| incorrectly |  |  | 2, intermixed | 62 | 0.571 | .57 |
|  |  | Image-word | 3 | 46 | 2.434 | .019* |
|  |  | learning | 4, blocked | 54 | 0.734 | .466 |
|  |  |  | 4, intermixed | 56 | 2.037 | .046* |
|  | Multiple-choice | Word-image | 1 | 57 | 3.177 | .002** |
|  |  | learning | 2, blocked | 59 | 4.807 | <.001*** |
|  |  |  | 2, intermixed | 62 | 5.484 | <.001*** |
|  |  | Image-word | 3 | 46 | -0.197 | .844 |
|  |  | learning | 4, blocked | 55 | -0.065 | .949 |
|  |  |  | 4, intermixed | 56 | 2.375 | .021* |
| Pretested items | Cued recall | Word-image | 1 | 57 | 4.351 | <.001*** |
| guessed |  | learning | 2, blocked | 59 | 3.061 | .003** |
| correctly |  |  | 2, intermixed | 58 | 2.152 | .036* |
|  |  | Image-word | 3 | 44 | 3.515 | .001** |
|  |  | learning | 4, blocked | 50 | 3.426 | .001** |
|  |  |  | 4, intermixed | 54 | 2.428 | .019* |
|  | Multiple-choice | Word-image | 1 | 56 | 5.087 | <.001*** |
|  |  | learning | 2, blocked | 59 | 7.514 | <.001*** |
|  |  |  | 2, intermixed | 61 | 3.231 | .002** |
|  |  | Image-word | 3 | 44 | 2.458 | .018* |
|  |  | learning | 4, blocked | 52 | 3.357 | .001** |
|  |  |  | 4, intermixed | 56 | 1.703 | .094 |
| *Note.*p* < .05,***p* < .01,****p* < .001. Shown are the results of paired *t*-tests (pretested vs. read) for both cued recall and multiple-choice tests for each experiment. These comparisons were made among pretested items guessed incorrectly (i.e. participants guessed incorrectly during the multiple-choice pretest) and among pretested items guessed correctly (i.e. participants guessed correctly during multiple-choice pretest). Results reaching significance show an advantage for pretesting over reading. | | | | | | |
